# Resting-state functional-MRI in iNPH: can default mode and motor networks changes improve patient selection and outcome? Preliminary report

**DOI:** 10.1186/s12987-023-00407-6

**Published:** 2023-01-26

**Authors:** Sara Fabbro, Daniele Piccolo, Maria Caterina Vescovi, Daniele Bagatto, Yan Tereshko, Enrico Belgrado, Marta Maieron, Maria Cristina De Colle, Miran Skrap, Francesco Tuniz

**Affiliations:** 1 Department of Neurosurgery, ASUFC “Santa Maria Della Misericordia”, Piazzale Santa Maria Della Misericordia 15, 33100 Udine, Italy; 2 Department of Neuroradiology, ASUFC “Santa Maria Della Misericordia”, Piazzale Santa Maria Della Misericordia 15, 33100 Udine, Italy; 3 Department of Neurology, ASUFC “Santa Maria Della Misericordia”, Piazzale Santa Maria Della Misericordia 15, 33100 Udine, Italy; 4 Department of Physics, ASUFC “Santa Maria Della Misericordia”, Piazzale Santa Maria Della Misericordia 15, 33100 Udine, Italy; 5 Department of Clinical, Diagnostic and Pediatric Sciences, University of Pavia, Via Alessandro Brambilla 74, 27100 Pavia, Italy

**Keywords:** CSF dynamics, Default-mode network, Motor network, Idiopathic normal pressure hydrocephalus, Tap test, Resting-state functional-MRI, Ventriculoperitoneal shunt

## Abstract

**Background:**

Idiopathic normal pressure hydrocephalus (iNPH) is a progressive and partially reversible form of dementia, characterized by impaired interactions between multiple brain regions. Because of the presence of comorbidities and a lack of accurate diagnostic and prognostic biomarkers, only a minority of patients receives disease-specific treatment. Recently, resting-state functional-magnetic resonance imaging (rs-fMRI) has demonstrated functional connectivity alterations in inter-hemispheric, frontal, occipital, default-mode (DMN) and motor network (MN) circuits. Herein, we report our experience in a cohort of iNPH patients that underwent cerebrospinal fluid (CSF) dynamics evaluation and rs-fMRI. The study aimed to identify functional circuits related to iNPH and explore the relationship between DMN and MN recordings and clinical modifications before and after infusion and tap test, trying to understand iNPH pathophysiology and to predict the best responders to ventriculoperitoneal shunt (VPS) implant.

**Methods:**

We prospectively collected data regarding clinical assessment, neuroradiological findings, lumbar infusion and tap test of thirty-two iNPH patients who underwent VPS implant. Rs-fMRI was performed using MELODIC-ICA both before and after the tap test. Rs-fMRI data of thirty healthy subjects were also recorded.

**Results:**

At the baseline, reduced z-DMN and z-MN scores were recorded in the iNPH cohort compared with controls. Higher z-scores were recorded in more impaired patients. Both z-scores significantly improved after the tap test except in subjects with a low resistance to outflow value and without a significant clinical improvement after the test. A statistically significant difference in mean MN connectivity scores for tap test responders and non-responders was demonstrated both before (*p* = 0.0236) and after the test (*p* = 0.00137). A statistically significant main effect of the tap test on DMN connectivity after CSF subtraction was recorded (*p* = 0.038).

**Conclusions:**

Our results suggest the presence of a partially reversible plasticity functional mechanism in DMN and MN. Low values compensate for the initial stages of the disease, while higher values of z-DMN were recorded in older patients with a longer duration of symptoms, suggesting an exhausted plasticity compensation. The standardization of this technique could play a role as a non-invasive biomarker in iNPH disease, suggesting the right time for surgery.

*Trial Registration* Prot. IRB 090/2021.

## Background

Idiopathic normal pressure hydrocephalus (iNPH) is a primitive, progressive and partially reversible form of dementia, characterized by a damage in the interaction between multiple brain regions [1]. The treatment relies on lumbar or ventriculoperitoneal shunts (VPS) implantation [1–8]. Clinical improvement after surgery varies from 33 to 84%, depending on the outcome assessment procedure [2], the time of assessment and the selection criteria for VPS implant [3–8]. Early treatment improves the outcome and suggests the importance of early and reliable diagnostic makers [9].

The international literature highlights the role of neuroradiological parameters and invasive procedures [10]. Beside the signs of ventriculomegaly, the responses to invasive procedures, such as cerebrospinal fluid (CSF) infusion and tap test, could represent sensitive prognostic markers for iNPH patients [11–15]. However, their low specificity induces a poor accuracy in predicting surgery response [11–15]. Moreover, because of the lack of accurate diagnostic and prognostic quantitative biomarkers, frequent presence of comorbidities, and limited understanding of the pathophysiology of the disorder [16–18], only 8% of iNPH patients receives a disease-specific treatment [11].

In recent years, the registration of blood oxygenation level-dependent (BOLD) signal fluctuations at rest in different areas of the brain has been used in the study of neurodegenerative disease [19–22]. Because it could be considered as a non-invasive technique, that measures the basal neuronal activity and the interdependence of different cerebral circuits, and patients are not required to perform demanding cognitive tasks [21], resting-state functional-magnetic resonance imaging (rs-fMRI) has been used in cases of dementia and cognitive impairment [19–22]. In iNPH subjects, functional connectivity alterations have been demonstrated in inter-hemispheric, frontal, occipital, default-mode network (DMN) and motor network (MN) circuits, although the findings have poor consistency [20–22].

Herein, we report our results in a cohort of thirty-two iNPH patients who underwent infusion and tap tests and rs-fMRI, focusing on the possible complementary role of these techniques in predicting the response to VPS implant and suggesting the right time for surgery. In particular, we would like to identify functional circuits related to iNPH and explore the relationship between DMN and MN recordings from Multivariate Exploratory Linear Optimized Decomposition into Independent Components Analysis (MELODIC-ICA) and clinical modifications before and after infusion and tap test, aiming to improve the knowledge about iNPH pathophysiology and to predict the best responders to VPS implant.

## Methods

From January 2018 to December 2020, clinical assessment, neuroradiological findings, lumbar infusion and tap test results of thirty-two patients with possible and probable iNPH [10] were prospectively reviewed. The radiological data of thirty healthy controls were also collected. All procedures contributing to this work complied with the ethical standards of the relevant national and institutional committees on human experimentation and with the declaration of Helsinki. The local ethics committee approved the study (RIF. Prot IRB: 090/2021), and informed consent was obtained from each subject.

### Clinical assessment

Basic demographic data on age and sex were recorded at the point of referral. iNPH patients underwent clinical and neuroradiological evaluations on three consecutive days. On the first and third days, they underwent neurological assessment and magnetic resonance imaging (MRI) acquisition, while CSF dynamics evaluation and tap test were performed on the second day. Our neurological protocol included the Movement Disorder Society unified Parkinson’s disease rating scale (MDS-UPDRS), time up and go (TUG), cognitive-time up and go (TUG-C), 10-m walking test (10MWT), Tinetti’s scale, frontal assessment battery (FAB) and iNPH grading scale (iNPH-GS) [10]. The same neurologist evaluated the patients both before and after the test to eliminate the inter-observer discrepancy. Moreover, to reduce any possible bias, the neurologist did not know the results of the infusion test. We considered significant an amelioration of at least of 10% in TUG, TUG-C or 10MWT after CSF drainage [10]. Because a positive response to the tap test was characterized by a significant amelioration in all the movement scales we used (TUG, TUG-C and 10MWT), in the paper we refer to TUG alone.

Six months after shunt implantation, we evaluated the clinical response to surgery, using the same scales we considered for the tap test.

Moreover, we considered the age of onset of the disease, the duration of symptoms and the presence of comorbidities, referring to the modified-frailty index-11 (mFI-11) [23, 24].

### Infusion and tap tests

The study of CSF dynamics was performed in all iNPH patients. With the subject placed in the lateral recumbent position, a Tuohy spinal needle was connected to a Möller Medical LiquoGuard 7 (Fulda, Germany) pressure monitor and fluid infusion system. After baseline CSF pressure was measured, saline solution was infused at a constant rate of 1.5 mL/min until a stable pressure plateau was reached. The resistance to outflow (R_out_) was calculated [10]. We considered a value of 12 mmHg/mL/min to distinguish a positive test from a negative one [10]. After the infusion test was completed, CSF was drained until the pressure reached a value of 0 mmHg.

### Neuroradiological investigation

All patients underwent pre- and post-test MRIs, while a single neuroradiological investigation was performed in healthy subjects. The studies were conducted using a Philips Achieva 3 T (Best, Netherlands) whole-body scanner using a SENSE-Head eight-channel head coil and a custom-built head restrainer to minimize head movements. The neuroradiological protocol included volumetric inversion recovery (IR) T1-weighted, sagittal turbo spin echo (TSE) T2-weighted, axial echo-planar imaging (EPI) diffusion-weighted, volumetric fluid-attenuated inversion recovery (FLAIR), rs-fMRI and BOLD EPI single shot images, with the following parameters: TR/TE = 2500/35 ms, flip angle = 90°, 32 axial slices interleaved ascending order, slice thickness = 3 mm, FOV = 240 mm, acquisition matrix = 128 × 128, 200 dynamics were collected, total duration was 8.32 min. On these sequences, Evans’ index (EI), callosal angle (CA) and disproportionately enlarged subarachnoid-space hydrocephalus (DESH) features were recorded [10].

### f-MRI data processing and analysis

During rs-fMRI acquisition, subjects were asked to relax, lie down at rest, closing their eyes, and trying not to think about anything; the lights in the scanner were dimmed to promote relaxation. Analysis was performed with Statistical Parametric Mapping-12 (SPM-12) and ICA as implemented in the MELODIC part of the FMRIB Software Library (FSL) (www.fmrib.ox.ac.uk/fsl) and CONN-fMRI FC toolbox (version 17e) [25]. All preprocessing steps were performed in FSL suite. They included motion correction using MCFLIRT, slice-timing correction, removal of non-brain structures, spatial smoothing with a Gaussian kernel of full width at half maximum of 5 mm, and high-pass temporal filtering equivalent to 100 s (0.01 Hz) [20]. Functional images were recorded on the skull-stripped structural images and on standard MRI brain. A single 4D dataset was created by temporal concatenation of the pre-processed functional images. The original dataset was decomposed using ICA to identify large-scale patterns of functional connectivity, allowing MELODIC tool to use ICA model. The ICA analysis was performed at single subject level using *variance-normalize time courses* option and a set of 80 components [26]. The ICA components was manually classified in order to remove noise due to physiological sources, movement and artifacts [27]. By an evaluating the spatial map, the time course and the power spectrum we performed the classification of noise components, which were manually labelled as “bad” and regressed out of the dataset [27]. A group ICA has been run on the single subject clean pre-processed 4D rs-data, all maps were thresholded using a mixture modeling, *p* = 0.5 [27]. To reduce the bias in networks identification, the components that most closely matched the DMN and the MN were selected using an automated two-step process called the “goodness-of-fit” approach. The standard DMN and MN templates were downloaded from the FMRIB website. The dual-regression approach was used to identify the difference in DMN and MN networks between groups [26]. The z-value identified the intensity of the signal of the spatial map network [25, 26, 28]. Because a large majority of papers refers to DMN modifications in iNPH patients [20–22], but the findings have poor consistency, we decided to study this network. Moreover, because iNPH can be considered also as a movement disorder [10], we investigated the MN.

### Cohort stratification

Based on post-tap test clinical modifications and R_out_ values, patients were divided into four groups. Subjects with R_out_ values > 12 mmHg/mL/min were considered in Group 1, if a positive tap test result was recorded, or Group 2, if it was not. Subjects with low R_out_ values were classified on the clinical modification after the tap test in Group 3 (statistically significant clinical amelioration) and Group 4 (no clinical improvement).

### Statistical analysis

The population was described using means or medians ± standard deviations and ranges for continuous variables and percentages for categorical variables. Data were tested for the normal distribution using the Shapiro–Wilk test and homogeneity of variance was assessed using Levene’s test. A Student’s t or Mann–Whitney *U* tests were used to compare continuous variables between groups, as appropriate. Differences between groups were determined using one-way and two-way analysis of variance (ANOVA,) followed by a Tukey’s honest significant difference (HSD) or Bonferroni-corrected post-hoc test when appropriate. All analyses were conducted using RStudio version 2022.7.0.548 (Boston, USA—http://www.rstudio.com). Relative statistical significance was set at *p* < 0.05.

## Results

Thirty-two patients were included. Table 1 summarizes their characteristics. The population was composed of 11 men (34.4%) and 21 women (65.6%), with a mean age of 73.9 ± 6.7 years-old (range 53–86). Eighteen women (60%) and twelve men (40%), with a mean age of 74.3 ± 7.2 years-old (range 56–86), were enrolled as healthy controls.

**Table 1:** Population characteristics

Parameter	Value
No. of patients	32
*Sex*	
Female	21 (65.6%)
Male	11 (34.4%)
Age at the referral	73.9 ± 6.7 years-old (range 53–86)
*Neuroradiological parameters*	
EI	0.38 ± 0.04 (range 0.30–0.47)
CA	96.3° ± 19.4° (range 63°-130°)
DESH	19 (59.4%)
*Infusion test*	
R_out_	11.52 ± 2.45 mmHg/mL/min (range 6.00–16.00)
*TUG*	
Pre-test value	18.34 ± 13.37 (range 10–65)
Post-test value	16.67 ± 12.14 (range 9–65)
Improvement (%)	7.89 ± 13.56 (range -25.71–34.78)
*MN*	
Pre-test value	11.44 ± 2.53 (range 7.37–17.01)
Post-test value	13.38 ± 2.95 (range 8.06–18.95)
Improvement (%)	19.92 ± 27.24 (range -25.67–91.06)
*DMN*	
Pre-test value	13.63 ± 3.74 (range 6.77–26.31)
Post-test value	16.46 ± 4.01 (range 7.54–30.81)
Improvement (%)	25.93 ± 34.40 (range −31.02–139.44)
VPS implantation	32/32 (100%)
Clinical Improvement post-VPS	26/32 (81%)

CA, callosal angle; DESH, disproportionately enlarged subarachnoid-space hydrocephalus; DMN, default-mode network; EI, Evans’ index; MN, motor network; R_out_, resistance to outflow; TUG, time up and go; VPS, ventriculoperitoneal shunt

### Cohort stratification

**Fig. 1:**
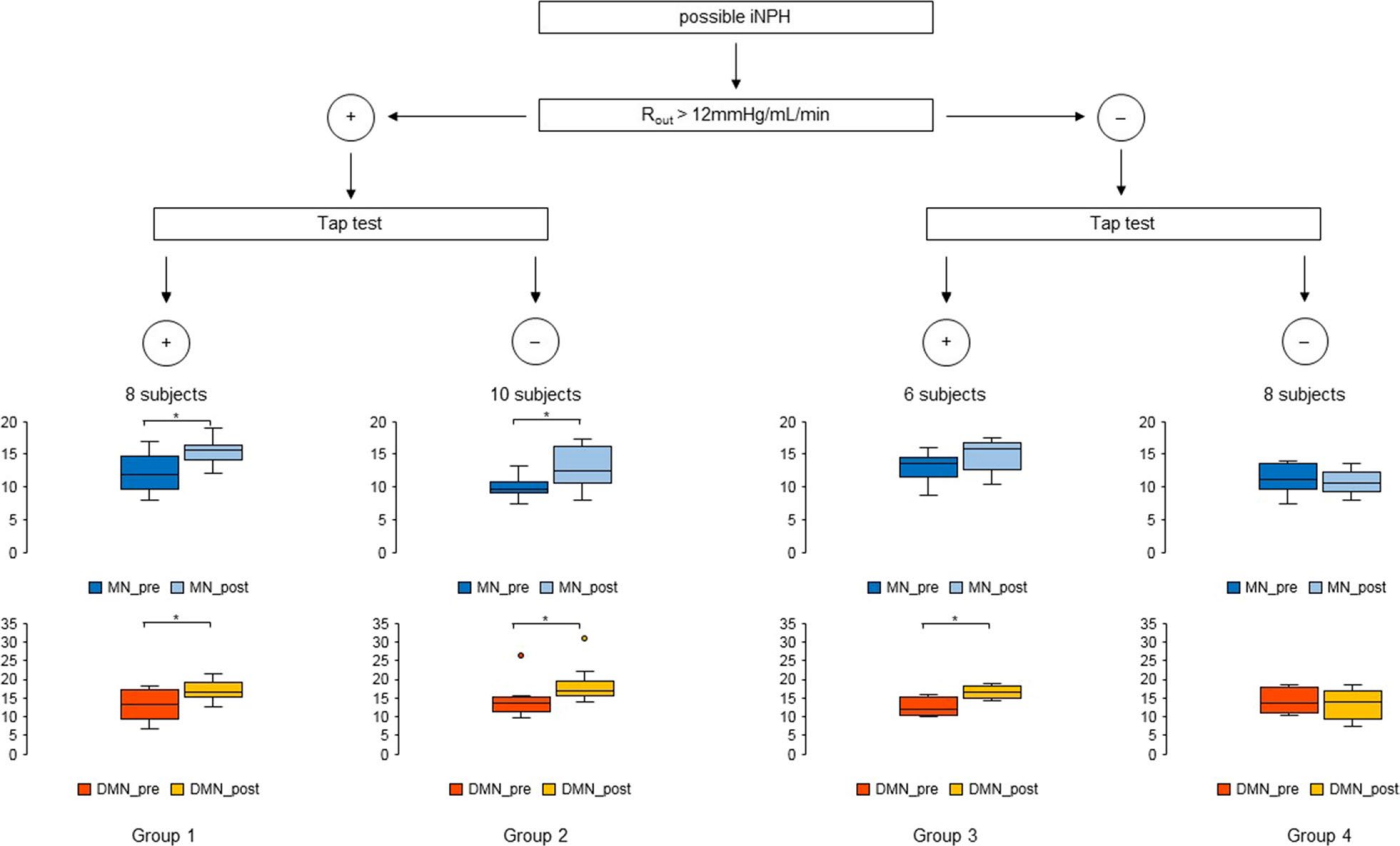
Cohort stratification. On the bases of CSF dynamics and tap test results, patients were classified in four groups. Eight subjects presented high R_out_ values and a positive response to the tap test (Group 1), while ten patients with positive CSF dynamics findings did not demonstrate clinical improvement after CSF subtraction (Group 2). Of those subjects with low R_out_ values, six were considered in Group 3 because of a good clinical response to the tap test, while eight patients did not improve in their presenting symptoms after CSF subtraction (Group 4). DMN: default-mode network; MN: motor network; R_out_: resistance to outflow; *p value < 0.05

On the bases of CSF dynamics and tap test results, patients were classified into four groups. In particular, eight subjects presented high R_out_ values and a positive response to the tap test (Group 1), while ten patients with positive CSF dynamics findings did not demonstrate clinical improvement after CSF subtraction (Group 2). Of those subjects with low R_out_ values, six were considered in Group 3 because of a good clinical response to the tap test, while eight patients did not improve in their presenting symptoms after CSF subtraction (Group 4). Figure 1 and Table 2 report the characteristics of each subgroup.

**Table 2:** Cohort stratification

	Group 1 (+/+)	Group 2 (+/−)	Group 3 (−/ +)	Group 4 (−/−)
No. of patients	8/32 (25%)	10/32 (31.25%)	6/32 (18.75%)	8/32 (25%)
*Sex*				
Female	4 (50%)	9 (90%)	4 (66.7%)	4 (50%)
Male	4 (50%)	1 (10%)	2 (33.3%)	4 (50%)
Mean age	73.6 ± 9.3 (range 53–85)	71.2 ± 5.7 (range 56–77)	74.5 ± 3.0 (range 71–79)	77.0 ± 5.3 (range 67–86)
Symptoms duration (months)	18.3 ± 2.7 (range 12–24)	21.6 ± 7.3 (range 12–30)	23.2 ± 6.8 (range 12–30)	37.8 ± 5.4 (range 30–48)
mFI-11	4.7 ± 1.1 (range 1–6)	5.2 ± 1.7 (range 0–7)	5.3 ± 2.9 (range 2–8)	7.8 ± 2.1 (range 4–10)
*Neuroradiological parameters*				
EI	0.39 ± 0.03 (range 0.33–0.45)	0.38 ± 0.05 (range 0.32–0.47)	0.36 ± 0.04 (range 0.30–0.43)	0.37 ± 0.04 (range 0.31–0.41)
CA	94.3° ± 24.1° (range 63°–130°)	95.0° ± 19.4° (range 65°–129°)	100.7° ± 19.4° (range 79°–130°)	96.9° ± 12.6° (range 79°–103°)
DESH	5 (62.5%)	8 (80%)	4 (66.7%)	2 (25%)
*Infusion test*				
Rout	13.20 ± 1.00 (range 12.00–15.00)	13.39 ± 1.29 (range 12.00–16.00)	9.56 ± 1.79 (range 6.00–11.33)	8.98 ± 1.43 (range 6.67–11.33)
*TUG*				
Pre-test value	22.56 ± 15.39 (range 13.0–61.0)	18.15 ± 15.75 (range 10.5–65.0)	17.33 ± 12.90 (range 10.0–46.0)	15.13 ± 3.77 (range 10.5–22.0)
Post-test value	18.81 ± 13.73 (range 9.5–54.0)	17.90 ± 15.87 (range 10.0–65.0)	12.67 ± 7.76 (range 9.0–30.0)	15.94 ± 4.68 (range 10.5–23.0)
Improvement (%)	17.47 ± 5.89 (range 10.71–26.20)	1.73 ± 6.97 (range −11.76–9.09)	22.21 ± 8.87 (range 10.00–34.78)	−4.73 ± 11.35 (range −25.71–8.33)
*MN*				
Pre-test value	12.24 ± 2.92 (range 7.94–17.01)	9.96 ± 1.65 (range 7.37–13.33)	13.04 ± 2.27 (range 8.66–16.09)	11.92 ± 2.10 (range 7.45–13.96)
Post-test value	15.38 ± 1.87 (range 12.18–18.95)	12.97 ± 2.96 (range 8.06–17.38)	14.95 ± 2.40 (range 10.38–17.54)	10.73 ± 1.70 (range 8.06–13.56)
Improvement (%)	30.73 ± 26.46 (range 7.81–91.06)	30.16 ± 21.14 (range 2.94–66.63)	15.28 ± 9.21 (range 4.40–31.99)	−0.20 ± 31.25 (range −25.65–59.19)
*DMN*				
Pre-test value	12.98 ± 3.97 (range 6.77–18.33)	14.24 ± 4.42 (range 9.78–26.31)	12.57 ± 2.29 (range 10.04–16.06)	14.30 ± 3.09 (range 10.40–18.56)
Post-test value	17.05 ± 2.67 (range 12.56–21.59)	18.31 ± 4.74 (range 14.02–30.81)	16.60 ± 1.59 (range 14.47–19.00)	13.44 ± 3.67 (range 7.54–18.59)
Improvement (%)	41.06 ± 39.92 (range 7.26–139.44)	32.56 ± 26.71 (range 2.57–86.07)	34.12 ± 11.34 (range 18.31–44.47)	−3.63 ± 30.71 (range -31.02–49.29)
*Surgery*				
VPS implantation	8/8 (100%)	10/10 (100%)	6/6 (100%)	8/8 (100%)
Clinical Improvement	8/8 (100%)	10/10 (100%)	6/6 (100%)	2/8 (25%)

CA, callosal angle; DESH, disproportionately enlarged subarachnoid-space hydrocephalus; DMN, default-mode network; EI, Evans’ index; mFI-11, modified-frailty index-11; MN, motor network; R_out_, resistance to outflow; TUG, time up and go; VPS, ventriculoperitoneal shunt. No statistical significant difference between neuroradiological parameters before and after the test was recorded. For this reason, in this table, we report only basal values

### Clinical evaluation

In the large majority of cases, patients referred gait impairment (31/32), while memory disorders and urinary incontinence were less frequent (20 and 25 subjects, respectively). The triad was complete in fourteen cases. Only two patients complained of gait imbalance as the sole clinical manifestation. No patient lamented urinary incontinence or memory disorders alone.

The results of neurological evaluation both before and after the test are reported in Tables 1 and 2. After the tap test, 14 patients demonstrated a significant amelioration in walking-tests (eight in Group 1 and six in Group 3), while 18 subjects did not improve in their presenting symptoms (ten in Group 2 and eight in Group 4). Moreover, two subjects in Group 2 and four in Group 4 presented a clinical worsening after the test.

Patients of Group 4 presented with the longest symptoms duration and the major number of comorbidities (Table 2). In particular, hypertension was recorded in all these subjects, while only two patients of this group did not present diabetes mellitus.

All patients underwent VPS implant. Six months after surgery, all subjects of Groups 1, 2 and 3 reported either improvement or halted progression in their presenting symptoms, with a statistically significant amelioration in neurological objective scales. Except for two cases, Group 4 did not demonstrate any clinical improvement after surgery. These two patients presented with the lowest DMN and MN pre-test scores and a significant amelioration in z-values was demonstrated after the tap test.

### CSF dynamics results

During the procedure, no complication was recorded. The mean R_out_ was 11.52 ± 2.45 mmHg/mL/min (range 6.00–16.00). High values of R_out_ defined Group 1 (13.20 ± 1.00 mmHg/mL/min) and Group 2 (13.39 ± 1.29 mmHg/mL/min), while low values were registered in Group 3 (9.56 ± 1.79 mmHg/mL/min) and Group 4 (8.98 ± 1.43 mmHg/mL/min).

### Neuroradiological findings

All patients had ventriculomegaly. Axial MRI demonstrated symmetrical dilatation of both lateral ventricles, with a mean EI of 0.38 ± 0.04 (range 0.30–0.47). The major ventricular dilatation was recorded in Group 1. On the coronal scans, the CA of the entire cohort ranged from 63° to 130° (mean 96.3° ± 19.4°), while in Group 3 the mean value was 100.7° ± 19.4°. DESH findings were recorded in 19 patients (five in Group 1, eight in Group 2, four in Group 3 and two in Group 4). Probably due to the low numerosity, we did not identify any significant difference between groups before the test. Moreover, no significant modification of these indexes was recorded after the tap test itself.

### rs-fMRI results

**Fig. 2:**
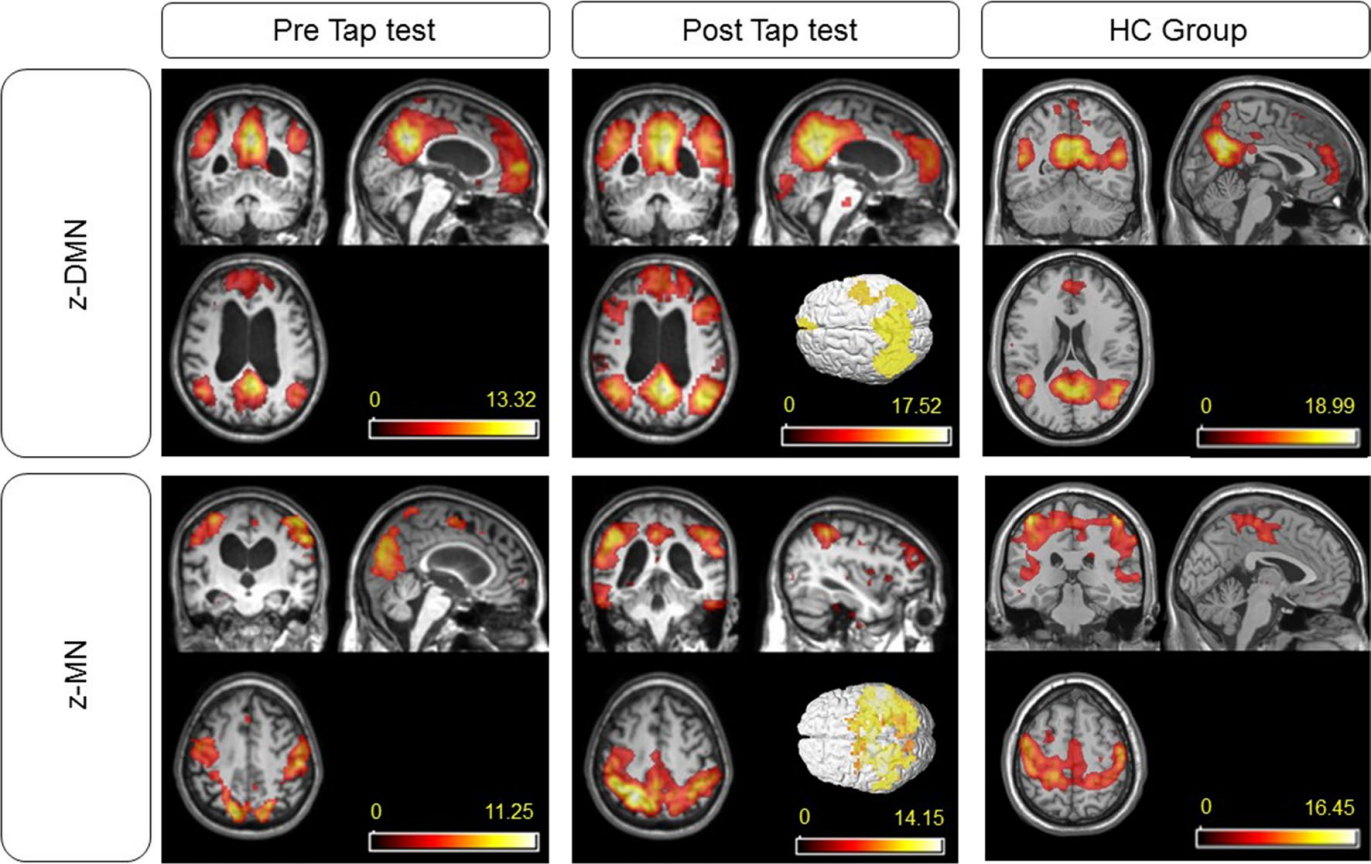
Comparison between patients with positive results after VPS implant and healthy controls. Z-DMN and z-MN improved after the tap test in iNPH subjects that favorably responded to VPS implantation. The values refer to the mean value of z-scores in Group 1, 2 and 3 and patients in Group 4 that improved after shunt implant. DMN: default-mode network; HC: healthy controls; MN: motor network

In iNPH patients, functional connectivity values were recorded both before and after the tap test. At the referral, the signal of the DMN and MN were significantly reduced compared to healthy subjects of similar demographic characteristics. After the test, we evidenced a statistically significant amelioration in the z-score of both parameters (*p* = 0.002 and *p* = 0.003, respectively), but the DMN and MN signals were significantly lower than those of the healthy population (Fig. 2). In healthy controls, the z-DMN and z-MN were 18.99 ± 1.67 and 16.45 ± 2.89, respectively.

We observed higher z-DMN and z-MN scores in patients with major clinical compromise and longer clinical history. A two-way ANOVA was conducted to examine the effects of the tap test and TUG score on DMN and MN connectivity. Residual analysis was performed to test for the assumptions of the two-way ANOVA. Normality was assessed using Shapiro–Wilk’s normality test and homogeneity of variances was assessed by Levene’s test. Residuals were normally distributed (*p* > 0.05) and there was homogeneity of variances (*p* > 0.05). There was a statistically significant main effect of TUG score on MN connectivity score both before (F(1, 28) = 5.736, *p* = 0.024) and after the tap test (F(1, 28) = 12.959, *p* = 0.001). Consequently, an analysis of simple main effects for TUG score was performed. There was a statistically significant difference in mean MN connectivity scores for tap test responders (n = 14) and tap test non-responders (n = 18) groups both before (*p* = 0.0236) and after the test (*p* = 0.00137). There was found also a statistically significant main effect of the tap test on DMN connectivity score after tap test (F(1, 28) = 4.732, *p* = 0.038).

**Fig. 3:**
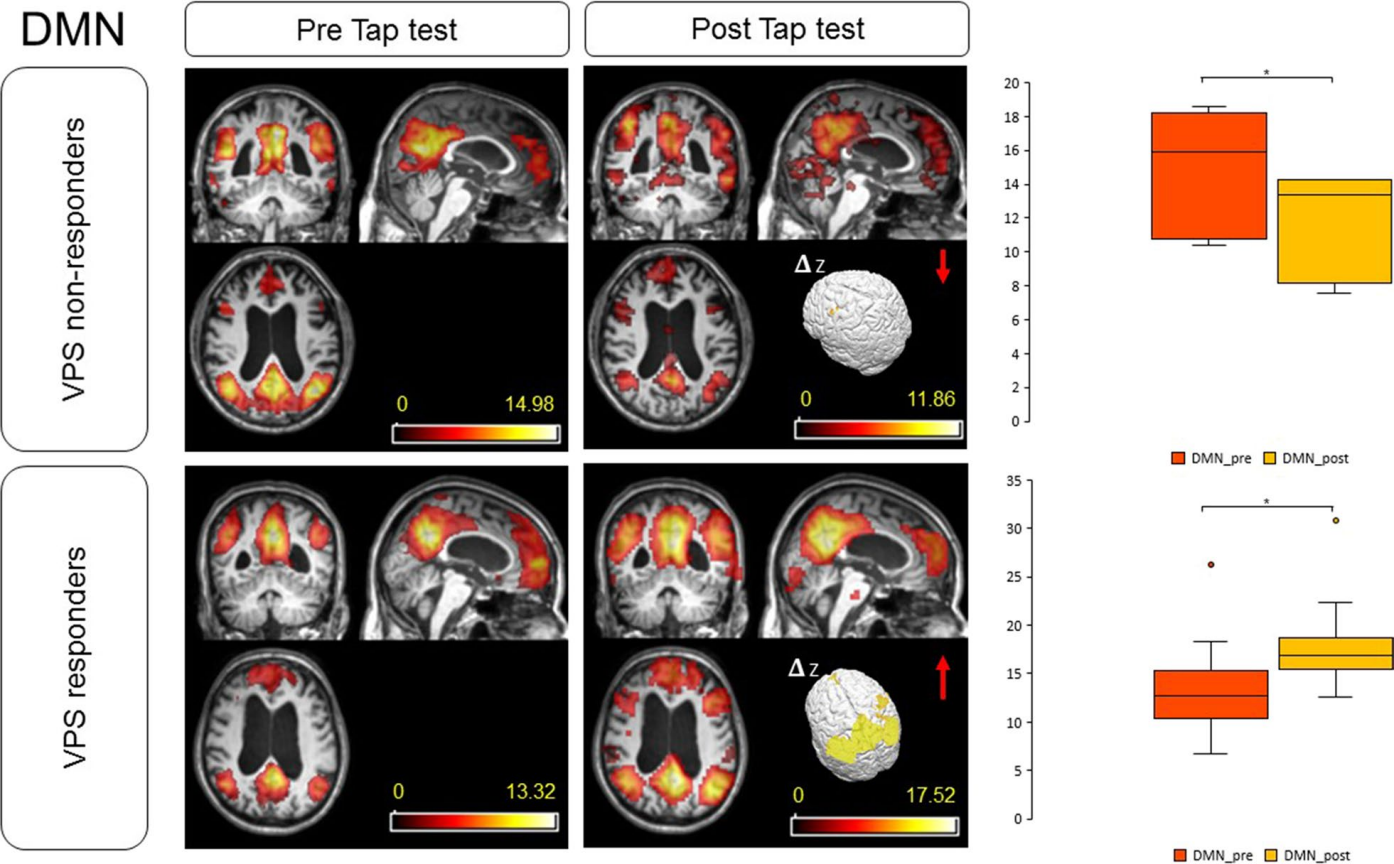
z-DMN modifications after the tap test. Herein, we compare the mean z-DMN between VPS responders (Group 1, 2, 3 and two patients of Group 4) and non-responders (the majority of Group 4). The score improved in responders, while decreased in non-responders. DMN: default-mode network; VPS: ventriculoperitoneal shunt. *p value < 0.05

**Fig. 4:**
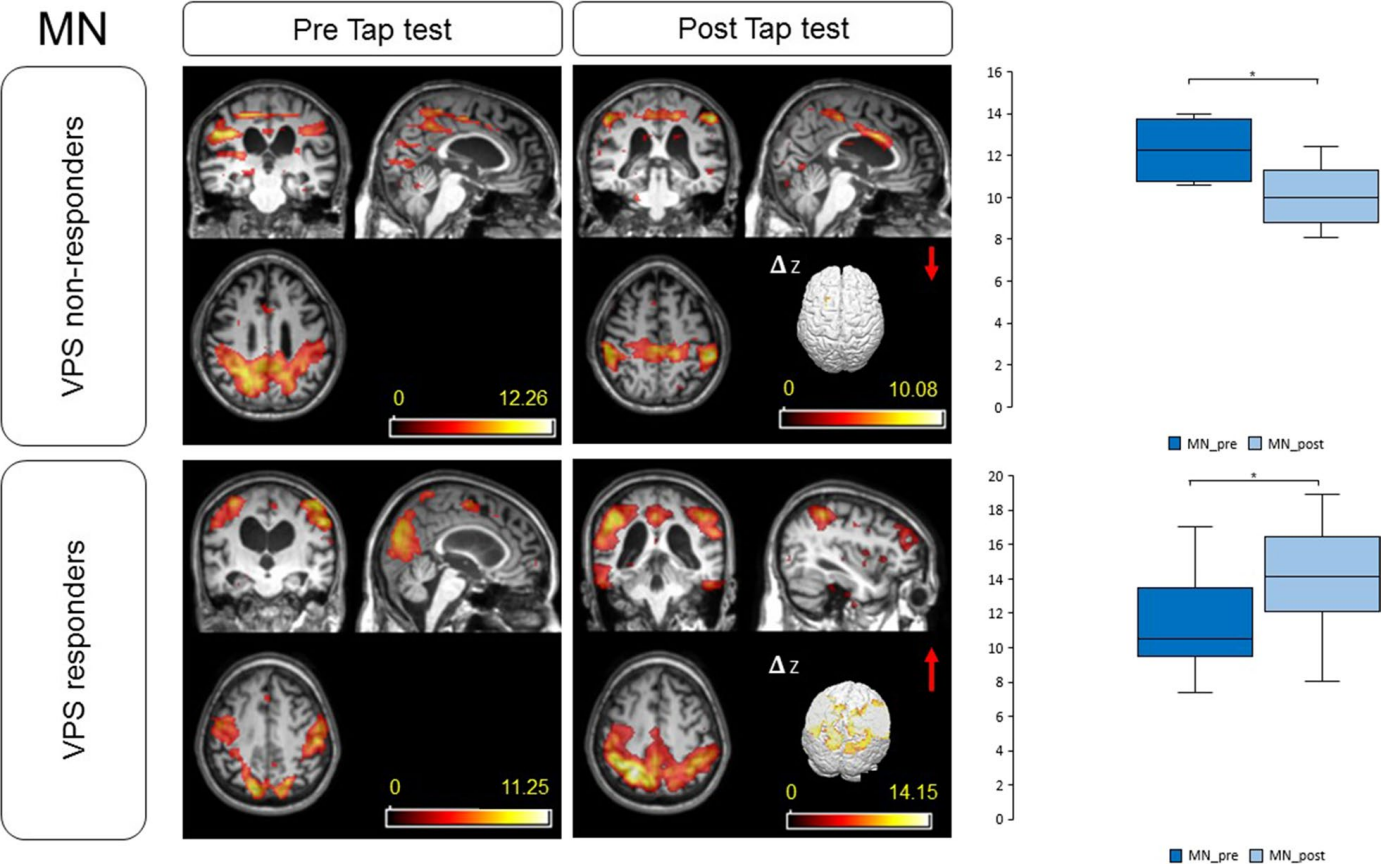
z-MN modifications after the tap test. Herein, we compare the mean z-MN between VPS responders (Group 1, 2, 3 and two patients of Group 4) and non-responders (the majority of Group 4). The score improved in responders, while decreased in non-responders. MN: motor network; VPS: ventriculoperitoneal shunt. *p value < 0.05

Analyzing the subgroups, higher values of DMN baseline signal were reported in Groups 2 and 4, in those patients who did not respond to the tap test. Higher baseline values of MN signal were recorded in Groups 1 and 3, in those patients who demonstrated a clinical improvement after the test. A higher percentage of z-DMN and z-MN score amelioration was reported in patients with higher values of R_out_ (Groups 1 and 2). After CSF removal, an amelioration in z-DMN and z-MN score was recorded in all patients, except those who did not improve after VPS implantation in Group 4 (Figs. 3, 4). These differences were statistically significant (*p* = 0.005 for DMN and *p* = 0.004 for MN, respectively). There was a significant difference between groups in MN connectivity score after the test as determined by one-way ANOVA (F(3, 28) = 5.616, *p* = 0.004). A Tukey post-hoc test revealed that the MN score after the test was statistically significant higher for patients in Group 1 (*p* = 0.004) and Group 3 (*p* = 0.019) compared to patients in Group 4.

No significant difference between groups was observed in DMN connectivity after the test (F(3, 28) = 2.543, *p* = 0.076).

## Discussion

iNPH should be considered a form of dementia, whose pathophysiology is associated with multiple reversible and irreversible neurobiological mechanisms [1, 10]. Neuroradiological investigations document a ventriculomegaly with mechanical compression and stretching of periventricular brain, accompanied by small blood-vessels damage and reduced blood flow in subcortical, frontal and temporal lobes, and decreased subcortical metabolism [1]. Altered CSF dynamics induce the development of interstitial edema and poor clearance of neurotoxic compounds in brain tissues [1]. Altogether, mechanical, vascular and CSF-flow mechanisms induce grey and white matter changes with subsequent damage in the connectivity between multiple brain regions [1].

A large population-based Swedish study reported the prevalence of iNPH to be around 6% of adults older than 80 years [11, 29]. Because of the presence of neurological mimics, the high prevalence of comorbidities and the absence of reliable predictors of shunt response [11, 29], only a minority of patients receives an accurate diagnosis and treatment [11]. The development of quantitative and accurate markers for iNPH diagnosis and prognosis is therefore needed [1].

### Clinical features, neuroradiological biomarkers and CSF dynamics

According to the international guidelines, the diagnosis of iNPH relies on the combination of brain imaging and clinical features [10]. In particular, gait disturbance is the most prominent symptom, occurring in 94 to 100% of patients, while the rates of cognitive impairment and urinary dysfunction vary from 78 and 60% to 98 and 92%, respectively [10]. In our population, gait imbalance was the predominant symptom (96.9%), while urinary disorders and cognitive impairment were less frequent (78.1% and 62.5%, respectively). The co-presence of all symptoms was recorded in 43.7% of our cohort, a lower value compared to the 60% reported in the literature [10].

The most important neuroradiological finding in iNPH is ventriculomegaly: an EI > 0.3 defines hydrocephalus, but ventriculomegaly also occurs in healthy elderly individuals [10]. DESH findings (ventriculomegaly, Sylvian fissure dilatation, and narrowing of the high convexity/midline subarachnoid spaces) are reported in 64% of patients, with a 77% positive and 25% negative predictive value [10]. Steeping of the CA indirectly expresses DESH and values < 90% are seen in most iNPH subjects [10]. It is useful to differentiate iNPH from Alzheimer’s disease (AD) and a cutoff of 90° demonstrates a positive predictive value of 93% [10]. Three-quarters of our population presented with DESH findings, while the CA ranged from 63° to 130°.

Because of the aging of the population and the augmented diagnosis of neurodegenerative disease, the recourse to invasive procedures (such as the study of CSF dynamics and physicochemical characteristics) has allowed differentiating iNPH from other forms of dementia [10]. The tap test has a sensitivity of 58% (26–87%) and a specificity of 75% (33–100%) [10]. Because of the high rate of false negatives (especially in patients with a long clinical story) [30], the tap test is generally accompanied by an infusion one. High values of R_out_ present a positive prediction rate of 80–92% relatively to VPS success [10].

### rs-fMRI in neurodegenerative disease

It is important to develop non-invasive and reliable diagnostic methods for iNPH [21]. Recently, rs-fMRI has attracted attention as a tool to help with clinical diagnosis and evaluation of neuropsychiatric and neurodegenerative disorders [19–22, 31–34]. Typical fMRI research focuses on the change in BOLD signal caused by the neural response to an externally controlled stimulus, while during rs-fMRI subjects are not required to perform demanding cognitive tasks [21]. Thus, rs-fMRI could be useful in cases of dementia and cognitive impairment [21]. It has been suggested that fluctuations in BOLD during rest reflect the neuronal baseline activity of the brain [31]. The statistical interdependence between signals recorded at different locations [32] represents patterns of synchronization and communication in the brain [33]. The DMN consists of discrete, bilateral and symmetric cortical areas [34] that are active during rest and suspended/deactivated when specific goal-directed behavior is needed [34–37]. While the ventral-medial prefrontal cortex supports emotional processing and the dorsal medial prefrontal cortex is associated with self-referential mental activity, the recollection of prior experiences is controlled by the posterior elements of the DMN (posterior cingulate cortex and the medial precuneus) [34]. At rest, the DMN demonstrated a baseline of fluctuating high activity and attenuation during particular tasks [31, 34]. In healthy people, an average percentage of BOLD signal fluctuation in the posterior cingulate and superior parietal areas of the DMN up to 3% has been described [31]. In AD, reductions in the DMN are mainly found in the posterior elements [20], with altered connectivity to the anterior ones (medial prefrontal cortex, anterior cingulate, superior temporal and inferior parietal region) [19]. rs-fMRI data present a sensitivity in classification of AD patients against healthy controls of 72–100% and specificity of 70–95% [19]. Unlike AD, no clear pattern of DMN is associated with Parkinson’s disease, while alteration of the motor and limbic connectivity seems to be a common pattern and the presence of hallucinations corresponds to a higher DMN activity with disruption of the connectivity between ventral and dorsal attention networks [19].

### rs-fMRI as an iNPH biomarker

It is demonstrated that, in iNPH, structural connectivity modifications affect periventricular white matter spanning (corticospinal tract [CST], corona radiata, superior thalamic radiation), homotopic connections (corpus callosum), the fronto-subcortical loops (anterior thalamic radiation), the frontoparietal (superior longitudinal fasciculus), and the parietotemporal (inferior longitudinal fasciculus) circuits [1]. These alterations converge in the frontal lobe and tap multiple cortical and subcortical areas; the anterior insula seems to be the intersection between locomotion, micturition and executive function meta-analyses [1, 21]. rs-fMRI studies evidenced an association between DMN and frontotemporal abnormal functional connectivity and cognitive and urinary, but not motor symptoms [20, 21]. Only Ogata found a relationship between urinary incontinence and functional connectivity in the cingulate, insula and frontal area [21].

Khoo reported the data of sixteen iNPH patients with improvement in their presenting symptoms after tap test [20]. Both region of interest (ROI)-based and voxel-based analyses revealed a reduced DMN connectivity [20]. In the voxel-based one, the DMN connectivity correlated positively with the iNPH-GS, as the cognitive and urinary continence domain scores, and negatively with the FAB score [20]. The significant peak in correlation was localized in the precuneus [20]. DMN connectivity and precuneus activity were hypothesized to decrease to compensate for impaired cognition, attention, gait and continence in patients with mild iNPH stage [20]. These results aligned with the observations of Miyazaki: the ^18^Fluoro-desossiglucose-positron emission tomography/computed tomography (FDG-PET/CT) scans revealed decreased brain glucose metabolism in the precuneus to posterior cingulate cortices in iNPH, but not in asymptomatic ventriculomegaly with features of iNPH on MRI (AVIM) [38]. The altered connectivity is not observed in preclinical stages, but only after symptoms onset [38]. With symptoms worsening, the compensatory decrease in DMN may not be maintained, resulting in an increase in DMN connectivity relative to that in the mild stage [20]. Similarly, our data demonstrated a higher baseline value of z-DMN in patients that did not respond to the tap test, suggesting a more compromised plasticity mechanism. In particular, in first stages of the disease, DMN activity decreases to compensate superior functioning. For this reason, we speculated that DMN scores were lower in Group 1 if compared with Group 4.

Ogata reported the data of nine subjects with a probable diagnosis of iNPH and two with a possible one [21]. Greater contribution of the interhemispheric connectivity than that of the intra-hemispheric one was reported for gait, cognition, and marginally for urinary symptoms [21]. The main contribution to functional connectivity modifications was identified in the areas that were located near the enlarged cerebral sulci (superior and middle temporal pole, insula, orbitofrontal cortex and medial frontal one) [21]. These findings support the hypothesis that enlarged ventricles stretch periventricular fibers and induce an abnormal functional connectivity [21]. The improvement of iNPH triad after VPS implantation could be explained by the reduced compression of the corpus callosum and the restoration of the interhemispheric functional connectivity [21].

Griffa, reporting the result of a cohort of twenty-one iNPH patients, concluded that iNPH is mainly characterized by abnormal cross-network dynamics involving the DMN, rather than by an intrinsic impairment of the DMN itself [22]. In particular, the analyses demonstrated an abnormal interaction between the precuneus and higher-order cortical regions, but not between the precuneus and other regions of the DMN [22]. The modified DMN-salience network (SAL) and DMN-executive-control network (ECN) interactions supported the observation of a partially influenced hierarchy between task-positive (ECN, SAL) and task-negative (DMN) networks [39]. Connectivity between higher-order systems tends to increase, whereas connectivity between lower-order systems tend to decrease [22]. Thus, an increased baseline activity in the frontal lobe of iNPH patients could be interpreted as a compensatory mechanism in response to motor and executive deficits [22].

Our results confirmed the presence of an altered DMN activity in iNPH patients, when compared to healthy subjects [20, 21]. On the bases of infusion test data and clinical response to tap test, we clustered patients into four groups. High values of R_out_ defined Group 1, if associated with a clinical response to the tap test, and Group 2, if there was not a clinical amelioration after CSF removal. Group 3 and 4 were characterized by low R_out_ values and response or no-response to the tap test, respectively. We confirmed an amelioration in DMN connectivity after CSF removal [22], and, for the first time, we demonstrated an augmented functionality of the MN after the procedure, confirming the presence of a functional plasticity mechanism 24 h after the tap test. This short-term plasticity effect could be ascribed to the reversal of subcortical chronic ischemia after CSF drainage, with consequent restoration of periventricular pathways [40]. Clinically, patients improved in gait and continence, but not in cognitive performances after the tap test, corroborating the thesis that resting-state functional dynamics may recover quicker than cognitive functions [22]. Moreover, we documented a correlation between high z-DMN scores at the baseline and the tendency not to respond to the tap test, while high z-MN values at the baseline were associated with clinical improvement after CSF subtraction. Moreover, a high percentage of z-DMN and z-MN score amelioration was observed in patients with high R_out_ values. The majority of patients who underwent VPS implantation demonstrated subsequent symptoms amelioration. In these cases, DMN and MN implemented activity after tap test predicted a positive response to CSF shunting. No significant amelioration in DMN and MN signal was observed in the majority of Group 4. In particular, subjects who improved after VPS implantation were those who presented an amelioration in z-scores after the tap-test (Fig. 3, 4). Group 4 patients who did not improve were those who demonstrated higher values in z-DMN and z-MN before the tap test and who worsened in these scores after the procedure. These subjects were the oldest of our series and symptoms duration was the longest one (Table 2). DMN basal activity was the highest of the cohort and no significant amelioration was evidenced after CSF drainage or VPS implant. Moreover, a z-DMN score deterioration after CSF subtraction was recorded. We could speculate that, in these cases, the long duration of the disease and the presence of comorbidities (hypertension and diabetes mellitus) were associated with irreversible damages, which did not respond to the test: these patients were the frailest of the cohort [23, 24, 41]. Independently of iNPH diagnosis, an association between cerebral elastance and frailty has already been reported, with an effect size comparable to that between frailty and age, the latter being the strongest risk factor for frailty [41]. These patients generally do not beneficiate from surgery and have the major surgical risk profile [23, 24]. Similarly in our population, subject were exposed to surgical risks, but did not beneficiate from the procedure.

In Group 1, we recorded a positive response to tap test, high R_out_ values and improvement in z-DMN and z-MN scores. Moreover, the values of z-DMN were the lowest of the entire cohort, suggesting a promptly responsive compensational mechanism. We could consider these patients as those who will most benefit from surgery, because of a baseline favorable condition.

Focusing our attention on the patients with equivocal results to CSF dynamics evaluations and tap test (Groups 2 and 3), we observed an improvement in DMN and MN connectivity, suggesting a restored functional connectivity and a partially reversible condition. All patients reported some degrees of clinical amelioration after VPS implantation. In addition, the only patient in Group 2 who demonstrated a clinical deterioration after the tap test underwent VPS implantation and the benefits of surgery were recorded six months later. In this case, rs-fMRI revealed a complementary role and allowed us to suggest surgery.

Because the natural course of iNPH consists of symptoms progression over time [9] and functional connectivity depends on clinical severity [20], rs-fMRI could be able to identify reversible and irreversible damage, thereby improving treatment selection.

### Limitations

The low numerosity of our population did not permit us to identify a statistically significant cut-off, which could play a role as a prognostic and predictive factor, distinguishing patients who could benefice from surgery and those who could not. Larger studies need to be performed to support the hypothesis of the presence of a real cut-off in z-MN and z-DMN scores.

Our results complement the role of invasive test data, but they cannot substitute for them so far. This is still one fundamental problem regarding the use of rs-fMRI as a biomarker in iNPH [19].

## Conclusions

iNPH is a form of dementia characterized by an impaired interaction between multiple brain regions. Neuroradiological parameters and CSF dynamics invasive evaluations demonstrate low accuracy in predicting surgery response. rs-fMRI highlights reversible connectivity alterations in DMN and MN. The standardization of this technique could play a role as a non-invasive biomarker in iNPH disease.

## Data Availability

Not applicable.

## Abbreviations

10MWT: 10-Meter walking test
ANOVA: Analysis of variance
AD: Alzheimer’s disease
AVIM: Asymptomatic ventriculomegaly with features of iNPH on MRI
BOLD: Blood oxygenation level-dependent
CA: Callosal angle
CSF: Cerebrospinal fluid
CST: Corticospinal tract
DESH: Disproportionately enlarged subarachnoid-space hydrocephalus
DMN: Default-mode network
EI: Evans’ index
ECN: Executive-control network
EPI: Echo-planar imaging
FAB: Frontal assessment battery
FDG-PET/CT: ^18^Fluoro-desossiglucose-positron emission tomography/ computed tomography
FLAIR: Fluid-attenuated inversion recovery
FSL: FMRIB Software Library
HSD: Honest significant difference
ICA: Independent component analysis
iNPH: Idiopathic normal pressure hydrocephalus
iNPH-GC: INPH grading scale
IR: Inversion recovery
MDS-UPDRS: Movement Disorder Society unified Parkinson disease rating scale
MELODIC: Multivariate Exploratory Linear Optimized Decomposition into Independent Components
mFI-11: Modified-frailty index-11
MN: Motor network
MRI: Magnetic resonance imaging
R_out_: Resistance to outflow
ROI: Region of interest
rs-fMRI: Resting-state functional-MRI
SAL: Salience network
SPM: Statistical Parametric Mapping-12
TSE: Turbo spin echo
TUG: Time up and go
TUG-C: Cognitive-TU
VPS: Ventriculoperitoneal shunt
